# CRELD2 is a novel modulator of calcium release and calcineurin-NFAT signalling during osteoclast differentiation

**DOI:** 10.1038/s41598-022-17347-0

**Published:** 2022-08-16

**Authors:** Adam Duxfield, Jennifer Munkley, Michael D. Briggs, Ella P. Dennis

**Affiliations:** International Centre for Life, Biosciences Institute, Newcastle University, Newcastle Upon Tyne, NE1 3BZ UK

**Keywords:** Bone development, Bone remodelling, Differentiation

## Abstract

Cysteine rich with epidermal growth factor (EGF)-like domains 2 (CRELD2) is an endoplasmic reticulum (ER) resident chaperone protein with calcium binding properties. *CRELD2* is an ER-stress regulated gene that has been implicated in the pathogenesis of skeletal dysplasias and has been shown to play an important role in the differentiation of chondrocytes and osteoblasts. Despite CRELD2 having an established role in skeletal development and bone formation, its role in osteoclasts is currently unknown. Here we show for the first time that CRELD2 plays a novel role in trafficking transforming growth factor beta 1 (TGF-β1), which is linked to an upregulation in the expression of *Nfat2*, the master regulator of osteoclast differentiation in early osteoclastogenesis. Despite this finding, we show that overexpressing CRELD2 impaired osteoclast differentiation due to a reduction in the activity of the calcium-dependant phosphatase, calcineurin. This in turn led to a subsequent block in the dephosphorylation of nuclear factor of activated T cells 1 (NFATc1), preventing its nuclear localisation and activation as a pro-osteoclastogenic transcription factor. Our exciting results show that the overexpression of *Creld2* in osteoclasts impaired calcium release from the ER which is essential for activating calcineurin and promoting osteoclastogenesis. Therefore, our data proposes a novel inhibitory role for this calcium-binding ER-resident chaperone in modulating calcium flux during osteoclast differentiation which has important implications in our understanding of bone remodelling and the pathogenesis of skeletal diseases.

## Introduction

Throughout life, bone is continually remodelled to maintain skeletal size, shape and integrity. Bone remodelling involves the highly coordinated interplay between bone-forming osteoblasts and bone-resorbing osteoclasts that function in a homeostatic environment, with osteoclastogenesis and bone resorption preceding osteoblastogenesis and bone formation^1^. It therefore comes as no surprise that dysregulation of this finely tuned process is associated with a range of pathological diseases including osteoporosis and osteopetrosis^2^.

Bone resorption is a multistep process beginning with the differentiation, polarisation and adhesion of multi-nucleated haematopoietic stem cell-derived osteoclasts to the bone surface. This process is tightly regulated by osteoblast-osteoclast crosstalk that occurs via several mechanisms^3^ including cell–cell/cell-bone contact and cytokines. Such osteoclastogenic cytokines include canonical (e.g. receptor activator of nuclear factor kappa-Β ligand (RANKL))^4^ and non-canonical (e.g. transforming growth factor-Beta (TGF-β))^5^ cytokines that function to upregulate the expression of transcription factors including nuclear factor of activated T cells 1 (NFATc1), considered to be the master regulator of osteoclast differentiation.

Inactive NFATc1 exists within the cytoplasm in a phosphorylated form. Upon RANKL stimulation it undergoes dephosphorylation (activation) by the calcium-dependant phosphatase, calcineurin, and subsequent nuclear translocation where it regulates genes associated with osteoclast differentiation and activity^4^. This process relies on a sustained cytoplasmic oscillation of calcium from the endoplasmic reticulum (ER), which has been linked to ER-stress and the evolutionarily conserved signalling cascade termed the unfolded protein response (UPR)^6^ that functions to maintain ER homeostasis. In response to calcium depletion and ER stress, sarco/endoplasmic reticulum calcium-ATPase (SERCA2b) pumps on the ER membrane are upregulated^7^ allowing an influx of calcium into the ER, regulating calcium flux and restoring ER homeostasis. Furthermore, ER stress has also been linked with an upregulation of ER-resident calcium binding chaperones including calreticulin^8^ and calnexin^9,10^, which have been shown to regulate ER calcium levels.

The cysteine rich with epidermal growth factor (EGF)-like domains (CRELD) family of proteins consists of two structurally related ER-resident proteins; CRELD1 and CRELD2^11^. The CRELD family are multi-domain proteins comprised of varying numbers of calcium-binding EGF-like domains, and a highly conserved region of tryptophan and glutamic acid residues (WE) domain^12^. During embryonic development CRELD1 expression is mostly localised to soft tissues and plays an important role during heart development with mutations in *CRELD1* shown to result in atrioventricular septal defects^13^. *CRELD2* is an ER-stress inducible gene^14^ expressed in the developing skeleton and has been implicated in the pathogenesis of skeletal dysplasias such as multiple epiphyseal dysplasia^15^ and metaphyseal chondrodysplasia type Schmid^16^, which are characterised by prolonged ER stress due to the retention and accumulation of mutant protein within the ER. To date the functions of CRELD2 have mainly been linked to protein folding and trafficking. For example, CRELD2 was first identified to be important in the folding and trafficking of the nicotinic acetylcholine receptor ɑ4 and β2 subunits^17^. In addition, an important role for CRELD2 has been identified as a protein disulphide isomerase (PDI)^18^ that functions in bone formation and homeostasis where it promotes the trafficking of the LRP1 receptor, subsequently modulating WNT signalling during chondrogenic and osteogenic differentiation^19^. Despite these recent studies linking CRELD2 to skeletal development and homeostasis, the role of CRELD2 still remains largely unknown and its role during osteoclastogenesis has never been studied. Therefore, the aim of this study was to understand the role of this novel chaperone protein in osteoclast differentiation.

## Results

### CRELD2 overexpression impairs osteoclast differentiation

**Figure 1:**
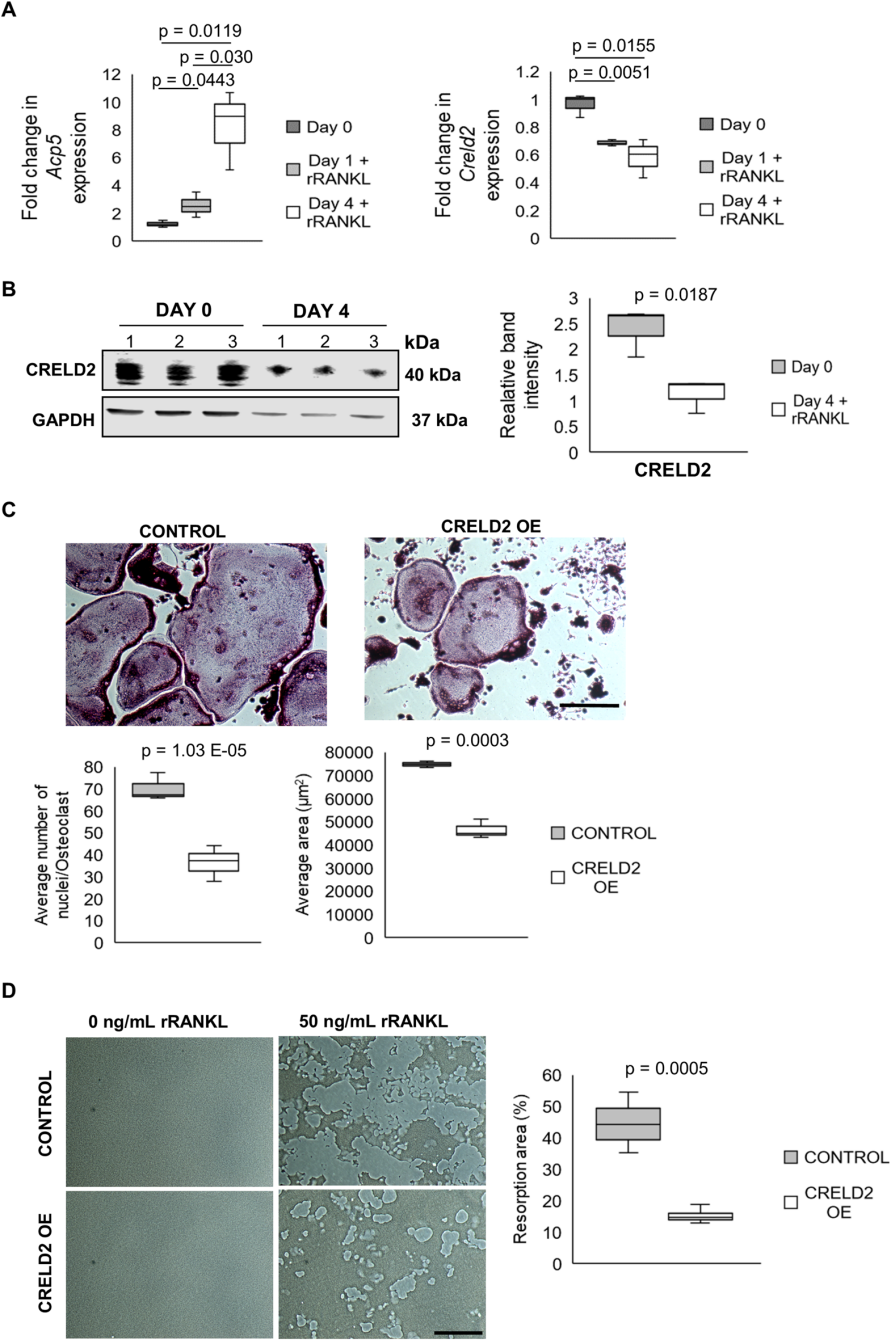
CRELD2 overexpression inhibits osteoclast differentiation. (**A**) qRT-PCR analysis of *Acp5* and *Creld2* expression in RAW264.7 cells pre and post treatment with recombinant RANKL (rRANKL). (n = 3 per condition). (**B**) Western blotting confirming CRELD2 is downregulated post treatment with rRANKL. (n = 3 per condition) (**C**) TRAcP staining and quantification of osteoclast area and number of nuclei/osteoclast 4 days after rRANKL stimulation. Scale bar = 200 µm. (n = 3 per genotype) (**D**) Resorption assay to assess osteoclast activity after 7 days stimulation with rRANKL. Scale bar = 200 µm. (n = 4 per genotype). Gene expression normalised to levels of *Actb.* Equal loading shown by GAPDH. Levels were determined by band densitometry. All graphs are displayed as mean ± standard deviation, significance denoted by p ≤ 0.05.

To study the role of CRELD2 in osteoclastogenesis, we first examined the expression of *Creld2* in RAW264.7 cells after osteoclastogenic stimulation. Interestingly, qPCR analysis revealed that the expression of *Acp5,* encoding the osteoclast marker enzyme tartrate-resistant acid phosphatase (TRAcP), was significantly upregulated 1 day and 4 days after RANKL stimulation indicating successful osteoclastogenesis; however, in contrast CRELD2 was significantly downregulated in RAW264.7 cells at the gene and protein level upon treatment with RANKL (Fig. 1A,B), consistent with findings that *Creld2* is downregulated following osteoclastogenic differentiation of mouse primary osteoclast precursors (Supplementary Fig. 1B). Despite playing important roles in promoting osteoblast and chondrocyte differentiation, we therefore hypothesise that the downregulation of *Creld2* may be required for osteoclast differentiation and that CRELD2 may inhibit early stages of osteoclastogenesis. Therefore, to study the role of CRELD2 in osteoclastogenesis, we knocked down *Creld2* expression during osteoclast differentiation of RAW264.7 cells using siRNA. Successful knockdown was confirmed by qPCR and western blotting (Supplementary Fig. 2A,B). Numerous studies have shown that osteoclastogenic differentiation is impaired if RAW264.7 cells become too confluent^20^ and here we show that CRELD2 ablation significantly increased the proliferation rate of RAW264.7 cells (Supplementary Fig. 2C,D). It is important to note that the cellular decision to exit proliferation and undergo differentiation is an important step in determining cell fate; however, we show that despite the increased proliferation of *Creld2* knockdown cells, *Creld2* ablation promoted cells to adopt an osteoclast fate (48 h after RANKL stimulation) as indicated by the upregulation of early osteoclast marker genes (Supplementary Fig. 3). Despite these findings, due to the increased proliferation of these ‘knockdown cells’ and in vitro osteoclast differentiation being negatively affected by cell confluency, we concluded that *Creld2* knockdown in RAW264.7 cells was not the appropriate model to study its role in osteoclastogenesis. Instead, we overexpressed CRELD2 (CRELD2 OE) in RAW264.7 cells (Supplementary Fig. 4A,B) and osteoclastogenesis was induced through RANKL supplementation for 4 days. Interestingly, overexpressing CRELD2 did not affect proliferation (Supplementary Fig. 4C), however, using a variety of techniques, we show that CRELD2 overexpression disrupts osteoclastogenesis. Staining for TRAcP, a histochemical marker of osteoclasts, revealed that CRELD2 OE osteoclasts were significantly smaller with significantly less nuclei/cell (Fig. 1B). In addition, this disruption in the differentiation of CRELD2 osteoclasts correlated with a decrease in resorption area of a synthetic crystalline calcium phosphate surface that mimics the bone surface (Fig. 1C).

**Figure 2:**
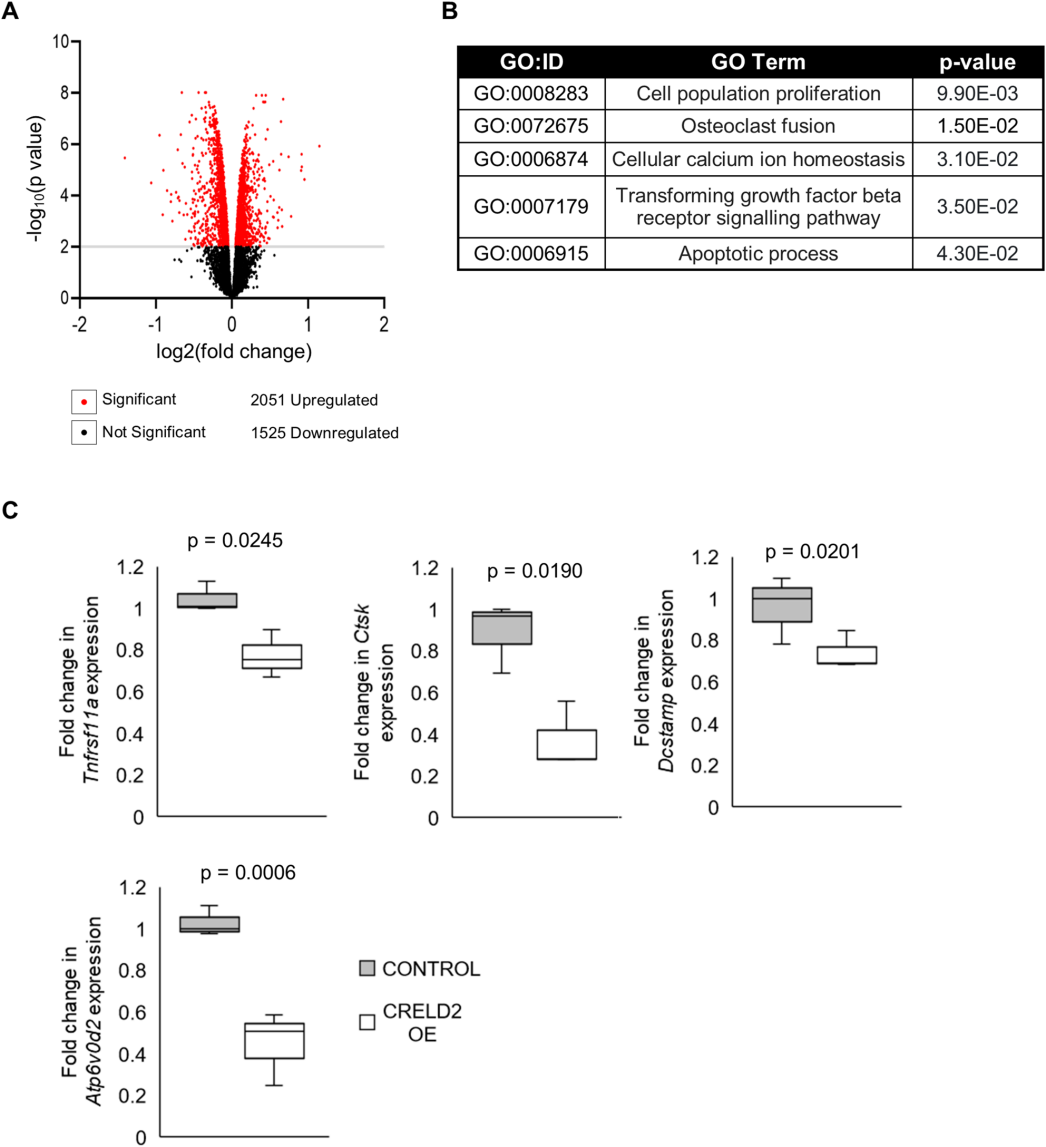
RNA-sequencing indicates that *Creld2* overexpression impairs osteoclastogenesis. (**A**) Volcano plot of differential gene expression between Control and OE osteoclasts at 2 days post rRANKL stimulation. Red dots indicate statistically significant (False discovery rate (FDR) adjusted *p* value ≤ 0.05, fold change ≥ 1.2 fold) differentially expressed genes and non-statistically significant genes are identified with a black dot. (**B**) Table of enriched Gene Ontology (GO) terms for the significant differentially expressed genes in CRELD2 OE osteoclasts (FDR adjusted p value < 0.05). (**C**) qRT-PCR analysis of key osteoclastogenic genes; *Tnfrs11a, Ctsk, Dcstamp, Atp6v0d2, Nfat2 and Ppp3ca* in control and CRELD2 OE osteoclasts 2 days post rRANKL stimulation. (n = 3 per genotype). Gene expression normalised to levels of *Actb.* All graphs are displayed as mean ± standard deviation, significance denoted by p ≤ 0.05.

To further assess the effects of CRELD2 overexpression on early osteoclast differentiation, we analysed the global gene expression profile of control and CRELD2 OE osteoclasts 48 h after RANKL stimulation by RNA-sequencing. CRELD2 overexpression resulted in the differential expression of 3576 genes (Fig. 2A). Gene Ontology (GO) term analysis highlighted clusters comprised of genes associated with ubiquitous cellular function as well as genes involved in osteoclastogenesis. The most notable clusters included genes associated with cell proliferation and apoptosis, the TGF-β signalling pathway and osteoclast fusion (Fig. 2B). The expression of several key osteoclastogenic genes were then validated by qPCR. For example, *Ctsk* encoding Cathepsin K a protease secreted by mature osteoclasts with an essential role in bone resorption^21^, was found to be significantly downregulated upon CRELD2 overexpression (Fig. 2C). In addition, genes encoding RANK the receptor for RANKL (*Tnfrsf11a*) and a transmembrane osteoclast fusion protein (*Dcstamp*)^22^ were also found to be significantly downregulated (Fig. 2C). Overall, these data suggest that CRELD2 overexpression impairs the process of osteoclastogenesis and disrupts osteoclast functionality.

### CRELD2 modulates *Nfat2* expression during the early stages of osteoclastogenesis through promoting TGF-β1 secretion

**Figure 3:**
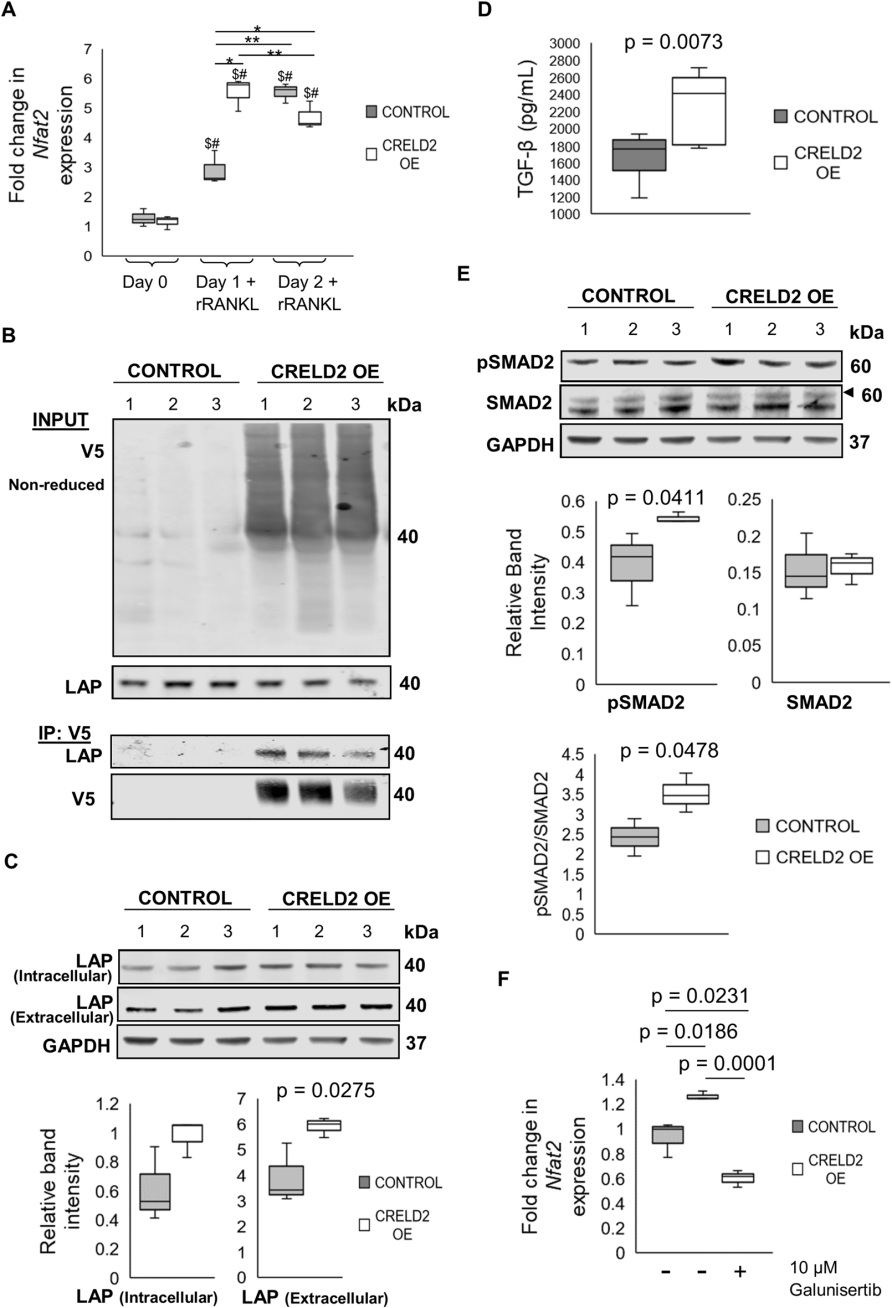
CRELD2 promotes TGF-β secretion. (**A**) qPCR-RT analysis of *Nfat2* expression in Control and CRELD2 OE osteoclasts during early stages of osteoclastogenesis. Osteoclast differentiation was successfully induced as *Nfat2* expression in Control osteoclasts was upregulated at day 1 (2.9 fold) and day 2 (5.5 fold) post RANKL treatment. $ = significant to Day 0 Control, # = significant to Day 0 CRELD2 OE. (**B**) Co-IP and western blot analysis showing the latent associated peptide (LAP) of TGF-β is pulled down with CRELD2-V5 in CRELD2 OE RAW264.7 cells indicating TGF-β is a binding partner of TGF-β. (**C**) Western blot showing the intracellular and extracellular expression of LAP. The levels of LAP in Control and CRELD2 OE osteoclasts were determined by band densitometry. (**D**) Concentration of active TGF-β in conditioned media from Control and CRELD2 OE osteoclasts measured using a luciferase reporter assay. (**E**) Western blot showing phospho-SMAD2 (pSMAD2) and SMAD2 expression in Control and CRELD2 OE lysates. (**F**) qPCR-RT analysis of *Nfat2* expression in Control and CRELD2 OE osteoclasts following treatment with the TGF-β inhibitor, Galunisertib. Gene expression normalised to levels of *Actb.* Equal loading shown by GAPDH. Levels were determined by band densitometry. n = 3 per genotype. Graphs displayed as mean ± SD, significance denoted by p ≤ 0.05.

Despite our results showing that CRELD2 overexpression inhibits osteoclastogenesis, we were surprised to see that the expression of the main transcription factor associated with osteoclastogenesis*, Nfat2* encoding NFATc1, was significantly upregulated in CRELD2 OE osteoclasts 24 h after RANKL stimulation (Fig. 3A). Although the expression of *Nfat2* was comparable 48 h after RANKL stimulation, our RNA sequencing results showed that genes regulated by NFATc1 were in fact significantly downregulated in CRELD2 OE osteoclasts. Since these findings did not correlate with the increased expression of *Nfat2* in CRELD2 overexpressing osteoclasts during early differentiation, we sought to understand the mechanism resulting in increased *Nfat2* expression in CRELD2 OE osteoclasts 24 h after RANKL stimulation and why this did not promote osteoclastogenesis.

In a recent study, we identified TGF-β1 as a CRELD2 binding partner^19^ in osteoblasts and chondrocytes. To study the binding partners of CRELD2 in RAW264.7 cells, co-immunoprecipitation was performed using a V5-antibody to bind V5-tagged CRELD2 in RAW264.7 CRELD2 OE cells as outlined previously^19^. Here, we show that CRELD2 also binds to TGF-β1 in RAW264.7 cells (Fig. 3B). Indeed, we show via western blotting that CRELD2 overexpression promoted the secretion of TGF-β1 into the media (Fig. 3C), which correlated with an increase in active extracellular TGF-β as determined using a luciferase reporter assay to measure bioactive TGF-β (Fig. 3D). This resulted in increased canonical TGF-β signalling in CRELD2 OE osteoclasts as identified by the upregulation of SMAD2 phosphorylation (Fig. 3E). A recent study found that TGF-β1 directly induces NFATc1 expression within 24 h of osteoclastogenic differentiation^23^. Here, we show that treating CRELD2 OE osteoclasts with the TGF-β inhibitor, Galunisertib, significantly reduced the increased *Nfat2* expression observed 24 h after RANKL stimulation (Fig. 3F), therefore, indicating that CRELD2 traffics TGF-β1 resulting in more active extracellular TGF-β, which in turn regulates *Nfat2* expression in the early stages of osteoclast differentiation.

### CRELD2 disrupts essential calcium release required for calcineurin/NFAT signalling during osteoclast differentiation

**Figure 4:**
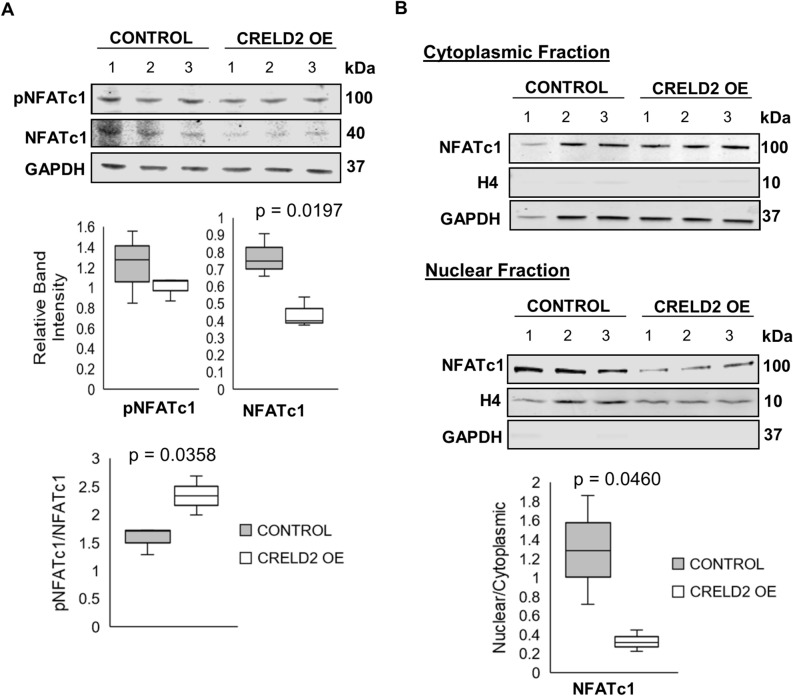
CRELD2 overexpression disrupts NFATc1 nuclear translocation during osteoclastogenesis. (**A**) Western blot of phospho-NFATc1 (pNFATc1) and total NFATc1 expression in Control and CRELD2 OE osteoclasts 24 h after rRANKL stimulation. Equal loading shown by GAPDH. Levels were determined by band densitometry. (**B**) Western blot showing the cytoplasmic/nuclear localisation of NFATc1 in Control and CRELD2 OE osteoclasts 24 h after rRANKL stimulation. GAPDH and Histone H4 (H4) were used as markers of the cytoplasmic and nuclear fractions respectively. Equal loading shown by GAPDH. Levels were determined by band densitometry. n = 3 per sample. Graphs displayed as mean ± SD, significance denoted by p ≤ 0.05.

The finding that CRELD2 overexpression resulted in an upregulation of *Nfat2* expression at Day 1 during osteoclastogenesis is contradictory to the impaired osteoclast differentiation and activity we observed (Figs. 1, 2). To further investigate the mechanism by which CRELD2 overexpression impairs osteoclastogenesis, we examined the expression of NFATc1 by western blotting. Although the expression of *Nfat2* was upregulated in CRELD2 OE osteoclasts 24 h after RANKL stimulation, we show that the protein levels of NFATc1 were in fact significantly downregulated (Fig. 4A). This resulted in an increased ratio of pNFATc1/NFATc1 in CRELD2 OE osteoclasts. Studies have shown that the phosphorylation status of NFATc1 is central to its activity; indeed, the dephosphorylation of NFATc1 by calcineurin A results in its translocation to the nucleus where it is active as a transcription factor. As we identified an increase in the levels of phosphorylated NFATc1 in CRELD2 OE osteoclasts, we then performed subcellular fractionation to isolate the nuclear and cytoplasmic compartments of control and CRELD2 OE osteoclasts. The subsequent western blot analysis of NFATc1 expression in these respective compartments showed that CRELD2 overexpression resulted in a reduced nuclear localisation of NFATc1 in CRELD2 OE osteoclasts (Fig. 4B).

**Figure 5:**
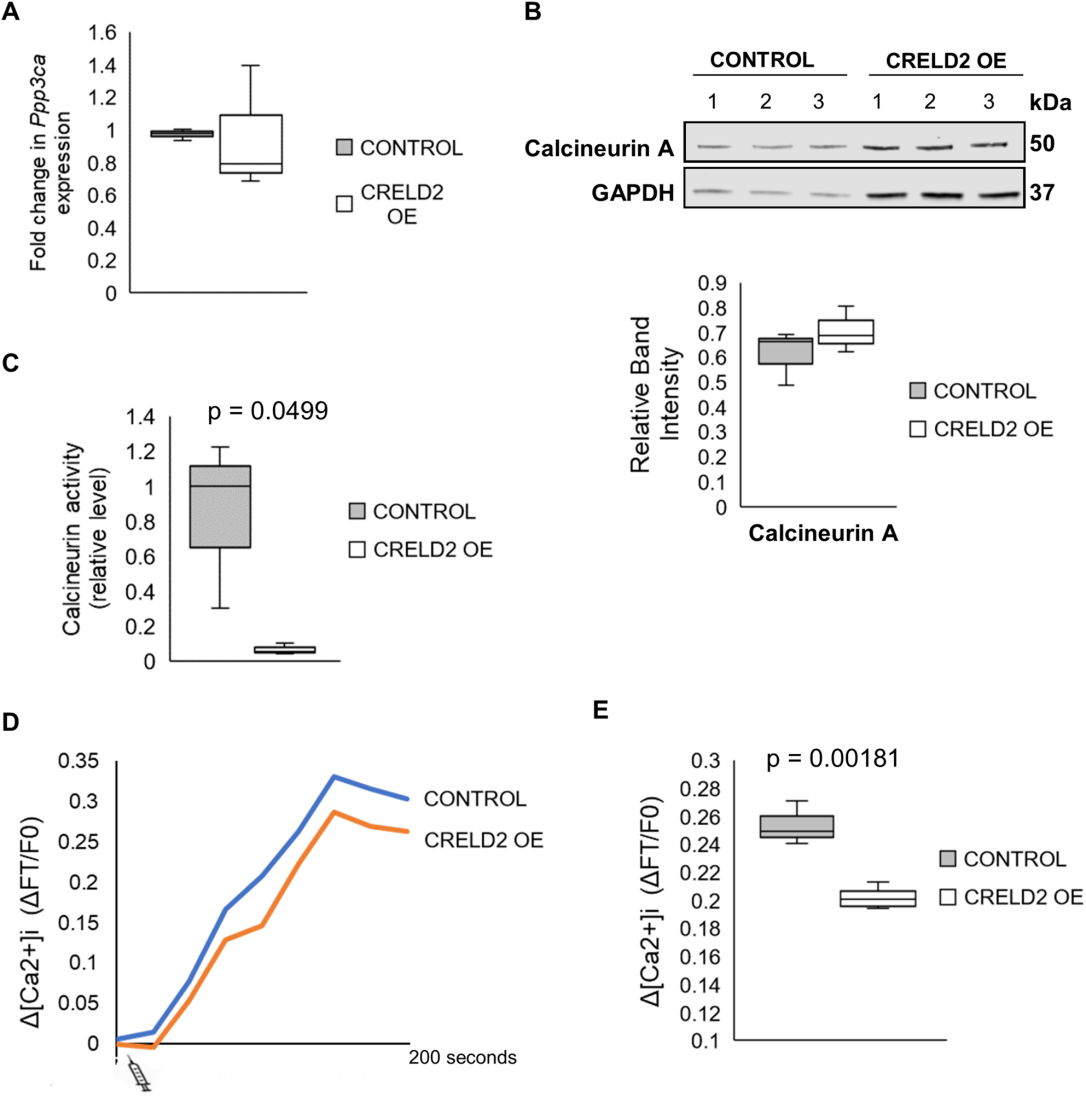
The overexpression of CRELD2 impairs calcium release and reduces Calcineurin activity essential for osteoclastogenesis. (**A**) qPCR-RT analysis and (**B**) Western blot showing Calcineurin A expression in Control and CRELD2 OE osteoclasts 24 h after rRANKL stimulation. (**C**) Cellular calcineurin phosphatase activity in Control and CRELD2 OE osteoclasts 24 h post rRANKL stimulation. (**D**) Calcium release from intracellular stores following thapsigargin treatment in control (Blue line) and CRELD2 OE (Orange line) RAW264.7 cells without RANKL stimulation. (**E**) Calcium release from intracellular stores in Control and CRELD2 OE RAW264.7 cells presented as change in fluorescence (ΔF) relative to basal fluorescence (F0). Gene expression normalised to levels of *Actb*. Equal loading shown by GAPDH. Levels were determined by band densitometry. n = 3 per sample. Graphs displayed as mean ± SD, significance denoted by p ≤ 0.05.

To further elucidate the mechanism by which CRELD2 overexpression impairs the nuclear translocation of NFATc1, we examined the expression and activity of calcineurin, a calcium-dependant phosphatase that dephosphorylates NFAT transcription factors. We found that the expression of the catalytic subunit of calcineurin (calcineurin A) was comparable at the gene and protein level between control and CRELD2 OE osteoclasts (Fig. 5A,B); however, the activity of calcineurin was significantly reduced following CRELD2 overexpression (Fig. 5C). Studies have shown that the activity of calcineurin is calcium-dependant and relies upon calcium release from the ER^24^. Here we show that calcium release is impaired in CRELD2 OE RAW264.7 cells, which could explain the reduction in calcineurin activity in CRELD2 OE osteoclasts (Fig. 5D,E). Therefore, our findings suggest that CRELD2 functions to modulate calcium release from the ER, subsequently regulating calcineurin activity and NFATc1 trafficking thereby having a detrimental impact on osteoclastogenesis.

## Discussion

A recent study has shown that CRELD2 plays an important role in skeletal development and bone homeostasis by promoting the differentiation of chondrocytes and osteoblasts^19^. Despite the well-established role of CRELD2 in bone-forming osteoblasts, its role in osteoclast differentiation is unknown. Since CRELD2 is expressed in the skeleton and plays a role in bone homeostasis, understanding its role in osteoclasts would provide further understanding of the complex process of bone remodelling, which would have an impact on our understanding of associated diseases.

Here, we show that CRELD2 expression is decreased upon stimulation of osteoclast differentiation. In addition, we show that overexpressing CRELD2 in RAW264.7 cells impairs osteoclast differentiation and activity as determined by a significant reduction in osteoclast size, number of nuclei and resorptive activity. This was confirmed by transcriptomic analyses that showed a downregulation in the expression of multiple osteoclastogenic marker genes in CRELD2 OE osteoclasts.

Although overexpressing CRELD2 reduced osteoclast differentiation of RAW264.7 cells, we were surprised to find that the expression of *Nfat2,* encoding NFATc1 the master regulator of osteoclastogenesis, was significantly upregulated in CRELD2 OE osteoclasts 24 h after RANKL stimulation. To understand how CRELD2 overexpression resulted in increased *Nfat2* expression, we referred to results from a previous study that identified putative CRELD2 binding partners in two independent cell types by co-immunoprecipitation. Of note, CRELD2 was found to bind to TGF-β1. Previous studies have shown that TGF-β1 plays a complex and controversial role in osteoclastogenesis depending on the model system. For example, in co-culture with osteoblasts TGF-β downregulates RANKL expression and inhibits osteoclastogenesis, whereas it has been shown to directly stimulate commitment and osteoclastogenic differentiation of RAW264.7 cells by upregulating RANK expression^5,25,26^. In addition, a study has shown that TGF-β directly upregulates *Nfat2* expression within 24 h of RANKL stimulation^23^. Here, TGF-β was shown to commit monocytes to the osteoclast lineage in early differentiation; however, it performs an inhibitory role in the latter stages of osteoclastogenesis^23^.

Since we confirmed CRELD2 bound to TGF-β1 in RAW264.7 cells and previous cellular functions of CRELD2 demonstrated a role in protein folding and trafficking, we hypothesised that CRELD2 promotes the secretion of TGF-β1 from RAW264.7 cells. Indeed, we show here for the first time that CRELD2 overexpression promoted the extracellular trafficking of TGF-β1 resulting in increased TGF-β signalling in CRELD2 OE osteoclasts. We therefore hypothesised that the upregulation of *Nfat2* expression in CRELD2 OE osteoclasts 24 h after RANKL stimulation was attributed to increased TGF-β secretion. Despite the initial increase in *Nfat2* expression, by 48 h post-RANKL treatment the levels of *Nfat2* were comparable between control and CRELD2 OE osteoclasts, further indicating that TGF-β1 functions to prime pre-osteoclasts during the early stages of differentiation.

Inactive NFATc1 exists in the cytoplasm in a phosphorylated form. To be active as a transcription factor it undergoes dephosphorylation by the calcium-dependant phosphatase calcineurin and is translocated to the nucleus where it regulates the expression of genes including those that drive osteoclastogenesis. Interestingly, a recent study has shown that the first member of the CRELD family of proteins, CRELD1, is a regulator of NFAT/Calcineurin signalling during heart development. Here, CRELD1 promotes NFATc1 dephosphorylation and its subsequent nuclear translocation by binding to and modulating calcineurin activity^27^. Based on these findings, we therefore analysed if CRELD2 played similar roles in NFATc1 activation and nuclear translocation to understand how osteoclastogenesis was impaired following CRELD2 overexpression despite the upregulation of *Nfat2* in CRELD2 OE osteoclasts in early differentiation. It is important to note that although the expression of *Nfat2* was upregulated in CRELD2 OE osteoclasts 24 h after RANKL stimulation, this was not reflected at the protein level. In fact, total NFATc1 levels were reduced following CRELD2 overexpression, which resulted in an increase in the ratio of phosphorylated NFATc1 to total NFATc1, indicating that the transcriptional activity of NFATc1 in CRELD2 OE was decreased. This can be attributed to a reduction in the activity of calcineurin following CRELD2 overexpression, which in turn resulted in a reduction in nuclear localisation of NFATc1. We therefore sought to understand how overexpression of this ER-stress inducible and ER-resident calcium-binding protein affected the activity of cytoplasmic calcineurin. Unlike CRELD1 that has been shown to bind the regulatory subunit of calcineurin, calcineurin B^27^, CRELD2 did not pull down with calcineruin^19^ therefore suggesting that CRELD2 does not modulate its activity by direct binding. Studies have shown that other calcium-binding ER-resident proteins regulate calcium mobilisation, which is required for the activation of calcineurin. Indeed, the ER-stress regulated calcium binding chaperones, calreticulin and calnexin, have been shown to modulate and reduce cytoplasmic calcium flux^8–10^. Indeed, calreticulin expression has been linked to reduced osteoclast differentiation^10^ due to impaired calcium flux. We therefore sought to investigate the effects of CRELD2 expression on calcium release in RAW264.7 cells. Similar to findings from other studies, our data showed that the overexpression of the calcium-binding ER-resident chaperone protein CRELD2 resulted in a reduction in the overall level of calcium released from the ER. Recent work has shown that CRELD2 interacts with the ER-resident calcium binding protein reticulocalbin (RCAN) 1, which mediates calcium-dependent cellular activities and inhibits calcium release from the ER through binding to the calcium channel inositol 1,4,5-trisphosphate (IP3) receptor type1 (IP3R1). Based on these recent findings, we hypothesise that CRELD2 mediates calcium flux via interactions with RCAN1; however, the relationship between these two calcium-binding ER proteins requires further study.

In summary, our results propose a novel inhibitory role of CRELD2 during osteoclastogenesis. We show for the first time that although CRELD2 promotes the trafficking of TGF-β1, osteoclastogenesis is disrupted by CRELD2 expression. We also demonstrated that CRELD2 expression blocks calcium release from the ER, impairing osteoclast differentiation due to reduced calcium-dependant calcineurin activity and the subsequent nuclear translocation of NFATc1, the master regulator of osteoclastogenesis.

Although no mutations have been identified in *CRELD2* to date, these data highlight a novel role for CRELD2 in osteoclastogenesis and indicates *CRELD2* may be a potential genetic locus for skeletal diseases caused by dysregulated osteoclast differentiation and function, such as osteogenesis imperfecta or osteoporosis. Such findings must be validated in vivo and could have important implications on our understanding of the complex process of bone degradation and remodelling in health and disease. We therefore propose that CRELD2 is a potential druggable target in osteoclast precursors which could modulate osteoclast differentiation and osteolysis in diseases characterised by defective osteoclast differentiation and/or function such as osteopetrosis and osteoporosis.

## Methods

### Cell culture and transfection

RAW264.7 murine monocyte/macrophage-like cells (ATCC, TIB-71) with the ability to differentiate into multinucleated osteoclasts, were maintained in complete α-MEM media supplemented with 10% FBS, 1 U/mL penicillin and 1 µg/mL streptomycin. To knockdown *Creld2* expression (siCRELD2), siRNA gene silencing was used as outlined previously^19^. To overexpress CRELD2 (CRELD2 OE) a previously generated^19^ V5-tagged wild-type *Creld2* cDNA construct in pcDNA3.1(+) was transfected into cells using Lipofectamine 2000 according to the manufacturer’s instructions. Empty pcDNA3.1(+) was expressed in Control cells.

### Analysis of cell proliferation

To analyse cell proliferation, cells were cultured in Amersham Cell Proliferation Labelling Reagent Bromodeoxyuridine (BrdU) for 2 h. Cells were then fixed in 4% paraformaldehyde for 10 min at room temperature and immunofluorescence was performed as outlined previously^19^ using an anti-BrdU antibody (ab6326, Abcam) to detect BrdU-positive cells. BrdU-positive cells were counted using the watershed algorithm in the FIJI program and expressed as a percentage of the total number of cells.

### Osteoclastogenesis

For osteoclastogenesis, control and *Creld2* knockdown/overexpression RAW264.7 cells were seeded at a density of 1.5 × 10^4^ cells/cm^2^ in osteoclastogenic media (complete α-MEM containing 50 ng/mL recombinant mouse RANKL (R&D Systems, 462-TEC-010/CF)). Osteoclastogenesis was performed by culturing RAW264.7 cells in osteoclastogenic media for 4 days, refreshing media after 2 days.

### RNA extraction and sequencing

RNA was extracted from cell pellets using the Promega ReliaPrep™ RNA Cell Miniprep System and the resulting RNA was sent to the Genomics Core Facility at Newcastle University for RNA sequencing. The samples were prepared using the Illumina Truseq stranded mRNA kit and analysed on an Illumina Nextseq 500 platform. The Galaxy open-source web-based platform for biomedical research^28^ was used to analyse the RNA sequencing results. This analysis included alignment of paired-end sequencing reads to the mouse (mm10) reference genome using the HISAT2. All adapter sequences were removed using the Cutadapt tool and SamTools-Merge was used to merge the output BAM files from the genome alignment for each sample. The number of reads which map to genes within the reference genome was determined using featurecounts. To identify the differentially expressed genes between the samples Limma-Voom was used, with gene annotations added using annotateMyIDs. The resulting output file showed both significant and non-significant differentially expressed genes, this gene list was filtered to show all biologically relevant fold changes (≤ 0.8 +  ≥ 1.2) with a p-value ≤ 0.05. RNA-sequencing hits were confirmed by quantitative-PCR (qPCR). The dataset generated in this current study is deposited on the Gene Expression Omnibus (GEO) repository, accession number GSE199189.

### Quantitative-PCR (qPCR)

cDNA was synthesised using the Script Reverse Transcription System according to the manufacturer’s protocol. RNA template was removed after incubation with 1 U RNase H for 20 min at 37 °C. qPCR was then performed using the POWER SYBR Green as stated in the manufacturer’s instructions using a Thermo Fisher QuantStudio 3 Real-Time PCR System. All primer sequences can be found in Supplemental Table 1. Samples were analysed in triplicate and expression was normalised to the level of *Actb.*

### Protein extraction and immunoblotting

Protein lysates were extracted and protein concentrations were determined as previously described^19^. Samples were denatured by boiling and reduced using Dithioreitol (DTT) where appropriate. For analysis of subcellular localisations, nuclear and cytoplasmic fractions were extracted using a Nuclear Extraction Kit (Abcam, Ab113474) as outlined in the manufacturer’s protocol. To study secreted protein expression, cells were cultured to confluency and media was replaced with serum free media. Cells were then cultured for a further 48 h in serum free media to allow proteins to build up in the conditioned media. Prior to western blotting, conditioned media was concentrated using Vivaspin 500 centrifugal concentrators according to manufacturer’s instructions. Western blotting was then performed as outlined previously^19^. In some cases, membranes were cut prior to probing with antibodies. The following primary antibodies were used: phosphoSMAD2 (#138D4), SMAD2/3 (#D7G7), Histone H4 (#2592) and NFATc1 (#D15F1) from Cell Signalling. Calcineurin A (ab52761) and TGF-β1 (ab215715) from Abcam. CRELD2 (HPA000603) from Cambridge Biosciences. GAPDH (mab374) from Sigma Aldrich, phosphoNFATc1 (Orb312385) from Biotbyt.

Membranes were imaged on the LI-COR Odyssey CLx Imaging System. Densitometric analysis of fluorescent protein bands were presented relative to GAPDH levels. Analysis was performed on 3 samples per genotype. Uncropped blots are shown in Supplementary Figures.

### Co-immunoprecipitation

RAW264.7 cells were transfected with a previously generated^19^ V5-tagged wild-type CRELD2 cDNA construct in pcDNA3.1 (+) to overexpress CRELD2 and an empty pcDNA3.1 (+) plasmid as a control. Co-immunoprecipitation was performed using anti-V5-agarose affinity gel (Abcam) to pull out V5-tagged CRELD2 and the resulting interacting partners as outlined previously^19^. CRELD2 interacting proteins were then identified by western blotting.

### Analysis of osteoclast differentiation and activity in vitro

Tartrate resistant acid phosphatase (TRAcP) staining was performed to analyse osteoclast number and area using the Acid phosphatase Leukocyte kit (Sigma Aldrich) according to the manufacturer’s protocol. The average osteoclast area and average nuclei number per osteoclast was determined using ImageJ. To measure the bone resorbing activity, a Corning^®^ Osteo Assay was used following the manufactures’ protocol. ImageJ was used to measure the percentage resorption area per well.

### Intracellular calcium release assay

To measure calcium release from intracellular stores, Control and CRELD2 OE RAW264.7 cells were loaded with the Rhod-4-AM fluorescent calcium probe for 45 min at room temperature to allow for dye esterification. Cells were washed in ice cold calcium-free Hank’s balanced salt solution supplemented with HEPES, pH7 and 5 mM EDTA to eliminate the effects of extracellular calcium influx. Calcium release from the ER was then measured using a FLUOstar OPTIMA fluorimeter after the addition of 2 µM thapsigargin in calcium-free buffer. For each assay, a 30 s baseline was established before the addition of thapsigargin and calcium release was displayed as the change in fluorescence/baseline fluorescence (ΔFT/F0).

### Calcineurin activity assay

To determine the activity of calcineurin in control and CRELD2 OE RAW264.7 cells, a Cellular Calcineurin Phosphatase Activity Assay Kit (Abcam) was used as outlined in the manufacture’s protocol.

### TGF-β activity assay

TGF-β activity was measured using the TGF-β luciferase reporter bioassay, a kind gift from Professor D. Rifkin (NYU), as outlined previously^29^. Luciferase activity was detected via the Luciferase Assay System (Promega) according to manufacturer's instructions.

### Statistics

All results are expressed as mean ± the standard deviation (SD) with three samples per experiment. A Student’s unpaired t-test was used to analyse statistical significance, with a significant value denoted by p ≤ 0.05.

## Supplementary Information

Supplementary Information 1.

Supplementary Information 2.

Supplementary Information 3.

Supplementary Information 4.

Supplementary Information 5.

Supplementary Information 6.

Supplementary Information 7.

Supplementary Information 8.

Supplementary Information 9.

Supplementary Information 10.

## Data Availability

All OMICs data generated from this paper will be shared with the scientific community by depositing on the publicly available database, GEOsets (accession number GSE199189).
