# Supplementary material for: CRELD2 is a novel modulator of calcium release and calcineurin-NFAT signalling during osteoclast differentiation
Source: Sci Rep. 2022 Aug 16;12:13884. doi: 10.1038/s41598-022-17347-0 (PMC9381524; doi:10.1038/s41598-022-17347-0)
Supplement: Supplementary file 10 — Supplementary Information 10. [file 41598_2022_17347_MOESM10_ESM.docx]

| **Gene** | **Species** | **Forward Primer** | **Reverse Primer** |
| --- | --- | --- | --- |
| *Creld2* | Mouse | 5’-GCAGAGAGCAGAAGGCAAA-3’ | 5’-GGATGCCCGTCACAAATC-3’ |
| *Tnfrsf11a* | Mouse | 5’-GGACAACGGAATCAGATGTGGTC-3’ | 5’-CCACAGAGATGAAGAGGAGCAG-3’ |
| *Nfat2* | Mouse | 5’-GGTGCCTTTTGCGAGCAGTATC-3’ | 5’-CGTATGGACCAGAATGTGACGG-3’ |
| *Dc-stamp* | Mouse | 5’-TTTGCCGCTGTGGACTATCTGC-3’ | 5’-GCAGAATCATGGACGACTCCTTG-3’ |
| *Atp6v0d2* | Mouse | 5’-ACGGTGATGTCACAGCAGACGT-3’ | 5’-CTCTGGATAGAGCCTGCCGCA-3’ |
| *Ctsk* | Mouse | 5’-AGCAGAACGGAGGCATTGACTC-3’ | 5’-CCCTCTGCATTTAGCTGCCTTTG-3’ |
| *Ppp3ca* | Mouse | 5’-CGGAAACCATGAATGTAGGCACC-3’ | 5’-GAAGGCATCCATACAGGCGTCA-3’ |

**Supplementary Table 1.** Primers used in Power SYBR Green qPCR reactions.
